# Sinomenine protects mice against *Staphylococcus aureus*-induced endometritis through inhibition of P2X7 receptor-mediated ferroptosis and inflammation

**DOI:** 10.1128/spectrum.03837-25

**Published:** 2026-06-30

**Authors:** Yue Zhang, Sixuan Li, Qingsong Sun, Musen Li, Linxiang Tang, Yunhe Fu, Chongshan Yuan

**Affiliations:** 1 Key Lab of Preventive Veterinary Medicine in Jilin Province, College of Animal Science and Technology, Jilin Agricultural Science and Technology University 381876, Jilin, China; 2 Department of Clinical Veterinary Medicine, College of Veterinary Medicine, Jilin University 623721, Changchun, Jilin Province, China; University of South Florida, Tampa, Florida, USA

**Keywords:** sinomenine, endometritis, ferroptosis, P2X7, inflammation

## Abstract

Endometritis is a major cause of infertility that adversely impacts both human reproductive health and livestock productivity. This study investigated the protective effects and underlying mechanisms of sinomenine (SIN) against *Staphylococcus aureus* (*S. aureus*)-induced endometritis. Seventy-two adult female mice were randomly divided into six groups: control group, SIN group (100 mg/kg), *S. aureus* group (1 × 10^7^ CFU per 200 μL PBS), and SIN (25 mg/kg, 50 mg/kg, and 100 mg/kg, respectively) + *S. aureus* groups. Uterine tissues were collected for analysis. Inflammatory factors, ferroptosis, and P2X7 signaling pathway-associated proteins were detected. *S. aureus* infection induced severe uterine pathology, significantly elevating myeloperoxidase (MPO) activity and pro-inflammatory cytokine levels (TNF-α, IL-1β, and IL-6; *P* < 0.05). Infection also triggered ferroptosis and upregulated P2X7 pathway components (*P* < 0.05). SIN administration dose-dependently attenuated these pathological changes, reversing endometritis severity (*P* < 0.05). These findings demonstrate that SIN alleviates *S. aureus*-induced endometritis by suppressing P2X7 receptor-mediated ferroptosis, providing a mechanistic rationale for its therapeutic potential.

**IMPORTANCE:**

We demonstrated for the first time that SIN could protect mice against *S. aureus*-induced endometritis. The mechanism was through inhibition of P2X7 receptor-mediated ferroptosis, which provides a theoretical basis for SIN treatment of endometritis.

## INTRODUCTION

The endometrium serves as the first line of defense within the uterine immune system, and its disruption compromises uterine resistance, predisposing the tissue to the development of endometritis (1). Endometritis can disrupt the estrus cycle, induce reproductive dysfunction, and ultimately result in long-term infertility in livestock, thereby inflicting substantial economic losses on the breeding industry (2). Current therapeutic strategies for endometritis primarily include uterine lavage, antibiotic therapy, and hormonal treatments; however, these approaches are often accompanied by challenges such as antimicrobial resistance and drug residues (3). Therefore, the identification of candidate compounds with minimal adverse effects from traditional Chinese medicine presents a promising alternative for the clinical management of endometritis in livestock. Ferroptosis, initially described in 2012 and formally defined by the Cell Death Nomenclature Committee in 2018 (4), represents a distinct form of regulated cell death characterized by two principal biochemical hallmarks: intracellular iron accumulation and lipid peroxidation (5). Glutathione peroxidase 4 (GPX4) functions as a critical negative regulator of ferroptosis by mitigating lipid peroxidation, thereby playing an essential role in maintaining cellular redox homeostasis (6). Accumulating evidence indicates that the activation of inflammation-related signaling pathways can lead to ferroptosis (7). In recent years, numerous therapeutic agents targeting inflammation and ferroptosis have demonstrated substantial efficacy in the treatment of tumor progression and ischemia-reperfusion injury (8). However, further investigation is required to elucidate the mechanisms of these agents in ferroptosis-associated inflammatory diseases to substantiate their clinical efficacy. P2XRs are expressed by several mouse and human immune cell types and can be divided into seven subtypes (9). Among them, the P2X7 receptor was initially identified in macrophages and lymphocytes (10), and it has been implicated in the regulation of systemic inflammatory signaling cascades (9). Activation of P2X7 has been shown to contribute to the pathogenesis of depression and rheumatoid joint symptoms (11, 12). Mechanistically, P2X7 receptor activation promotes the release of pro-inflammatory cytokines such as IL-1β and IL-18 by activating NLRP3 inflammasomes, thereby amplifying the inflammatory response (13). On the contrary, pharmacological inhibition of P2X7 has been reported to reverse these pathological effects (14).

*Sinomenium acutum* has been utilized in traditional Chinese medicine for centuries, primarily for its therapeutic efficacy against rheumatoid arthritis (15). Sinomenine (SIN), one of the main bioactive alkaloids derived from *S. acutum*, exhibits a broad spectrum of pharmacological effects, including immunomodulatory (16), antioxidant (17), sedative-analgesic (18), and anti-inflammatory effects (19). These diverse bioactivities position SIN as a promising candidate for clinical applications, particularly in the management of inflammatory conditions. Mechanistic studies have demonstrated that SIN attenuates inflammation and oxidative stress in female rats through regulation of the TLR4/MyD88/NF-κB signaling pathway (20). In addition, SIN exerts protective effects against DSS-induced intestinal inflammation *via* inhibition of NF-κB/p65 signaling (21). Although SIN has been extensively investigated as a bioactive compound with anti-inflammatory properties, its underlying mechanisms of action remain incompletely elucidated. Therefore, the present study aimed to explore the protective effects of SIN against endometritis and to elucidate the involvement of ferroptosis and the P2X7 receptor in its therapeutic action. By delineating the role of these pathways in the pathogenesis of endometritis, this research seeks to provide potential therapeutic strategies for the clinical management of this condition. Furthermore, these findings may contribute to a deeper understanding of the molecular mechanisms underlying SIN-mediated anti-inflammatory effects and offer insights into the mode of action of other classical anti-inflammatory agents.

## MATERIALS AND METHODS

### Animals

Seventy-two adult female BALB/c mice (aged 8–10 weeks; weighing 25–30 g) were obtained from the Center of Experimental Animals at the Norman Bethune Health Science Center of Jilin University (Jilin, China). The animals were housed under controlled conditions at 20–25°C with 40%–60% relative humidity and a 12-h light/dark cycle. All experimental procedures involving animals were performed in accordance with the guidelines of the Jilin University Institutional Animal Care and Use Committee and were approved by the Animal Ethics Committee of Jilin University.

### Endometritis model administration and treatment

Seventy-two mice were randomly assigned to six groups (*n* = 12 per group): a control group, a SIN group (100 mg/kg), a *Staphylococcus aureus* (*S. aureus*) group (1 × 10^7^ CFU in 50 μL PBS), and three SIN + *S. aureus* groups receiving 25, 50, or 100 mg/kg SIN, respectively. SIN (purity ≥98%) was purchased from Shanghai Yuanye Bio-Technology Co., Ltd. (Shanghai, China). To induce endometritis, *S. aureus* (1 × 10^7^ CFU in 50 μL PBS) was injected into each uterine horn using a 100 μL syringe fitted with a 30-gauge blunt needle, as previously described (22). The mice received *S. aureus* in the uterus for 24 h, as described previously (23). Upon successful establishment of the model, mice in the SIN + *S. aureus* and SIN groups were administered SIN solution at doses of 25, 50, or 100 mg/kg/d via intragastric injection for 7 consecutive days, based on a previous report (21). The control group received an equivalent volume of physiological saline.

### Tissue ferrous levels

Tissue ferrous levels were measured using a commercial kit (Beckman Coulter Life Sciences, Brea, CA, USA). Six animals from each group were randomly selected as biological replicates. Microtiter plates were coated with 100 μL per well of capture antibody diluted in 1 × Coating Buffer A and incubated overnight at 4℃. Following four washes with Wash Buffer, nonspecific binding sites were blocked by adding 200 μL per well of 1 × Assay Diluent A and incubating for 1 h at room temperature with continuous shaking. The plates were then washed four times, and 100 μL per well of either serially diluted standards or experimental samples were added, followed by incubation for 2 h at room temperature. After four additional washes, 100 μL per well of biotinylated detection antibody was introduced and incubated for 1 h under the same conditions. The plates were again washed four times, and 100 μL per well of avidin-HRP conjugate was added and incubated for 30 min at room temperature. A final series of five washes was performed, incorporating a 30–60 s soak during the last wash to minimize background. Subsequently, 100 μL per well of TMB Substrate Solution D was added, and plates were incubated in the dark for 15 min at room temperature to allow color development. The enzymatic reaction was quenched by adding 100 μL per well of Stop Solution, and absorbance was measured at 450 nm and 570 nm within 15 min using a microplate reader.

### Myeloperoxidase (MPO) activity detection

Six animals from each group were randomly selected as biological replicates. Uterine tissue was accurately weighed and fixed in extraction buffer to obtain a 5% homogenate. According to the manufacturer’s instructions, an MPO detection kit (Nanjing Jiancheng Biotechnology Research Institute, Nanjing, China) was used to detect MPO activity in the homogenate. Samples and serially diluted standards were dispensed into antibody-precoated microplates, and MPO antigen was captured during incubation. Following sequential washes, biotinylated detection antibody and horseradish peroxidase (HRP)-conjugated streptavidin were added stepwise, with unbound materials removed by extensive washing. Color development was initiated by adding TMB substrate, and the reaction was quenched with sulfuric acid. Absorbance was measured at 460 nm.

### Hematoxylin and eosin (H&E) staining

Six animals per group were randomly selected as biological replicates (*n* = 6). Uterine tissue samples were fixed in 10% neutral-buffered formalin for 24 h, followed by paraffin embedding. The embedded tissues were continuously sectioned at a thickness of 3–5 μm. Sections were deparaffinized in xylene for 5–10 min and rehydrated through a graded ethanol series (100%, 95%, 85%, and 70%) for 5 min each. Subsequently, sections were stained with hematoxylin solution for 5–15 min, rinsed thoroughly, and then counterstained with eosin solution for 1–5 min. After staining, the sections were dehydrated through graded ethanol (70%, 85%, 95%, and 100%) and cleared in xylene twice (10 min each). Finally, the sections were mounted with neutral resin and examined under an optical microscope (Olympus, Tokyo, Japan).

### Enzyme-linked immunosorbent assay (ELISA)

The levels of IL-1β, IL-6, TNF-α, MDA, ATP, and GSH in uterine tissue were measured using commercial enzyme-linked immunosorbent assay (ELISA) kits (Nanjing Jiancheng Bioengineering Institute, Nanjing, China) according to the manufacturer’s instructions. Six animals per group were randomly selected as biological replicates for these assays. Briefly, uterine tissue samples were homogenized in ice-cold normal saline and centrifuged at 12,000 rpm for 15 min at 4°C to obtain a 10% (w/v) tissue homogenate supernatant. The samples were added to pre-coated wells, followed by sequential addition of detection antibody, avidin-HRP conjugate, Substrate Solution D, and Stop Solution according to the manufacturer’s protocol. The optical density was measured at 450 nm using a microplate reader, and sample concentrations were calculated by interpolation from standard curves.

### Protein preparation and western blot analysis

Six animals per group were randomly selected as biological replicates for western blot analysis. Uterine tissue samples (0.05 g) were homogenized in 300 µL of tissue lysis buffer, followed by centrifugation at 12,000 rpm for 15 min at 4°C to collect the supernatant. Protein concentrations were determined using a BCA Protein Assay Kit (Beyotime Institute of Biotechnology, Shanghai, China). Equal amounts of protein (40 μg per lane) were loaded onto 10% sodium dodecyl sulfate-polyacrylamide gel electrophoresis (SDS-PAGE) gels. Proteins were separated electrophoretically at a constant voltage of 80 V for 35 min, followed by 120 V for 45 min. Subsequently, the separated proteins were transferred onto polyvinylidene fluoride (PVDF) membranes (Bio-Rad, Hercules, CA, USA) at 170 V for 1 h and 40 min. The membranes were blocked with 5% skim milk in tris buffered saline with tween 20 (TBST) for 3 h at room temperature and then incubated overnight at 4°C with specific primary antibodies against GPX4, SLC7A11, NF-κB p65, NF-κB p-p65, IκBα, p-IκBα, NLRP3, ASC, caspase-1, and P2X7. All primary antibodies were mouse monoclonal antibodies purchased from Cell Signaling Technology Inc. (Beverly, MA, USA). Following primary antibody incubation, the membranes were washed three times with TBST and incubated with horseradish peroxidase (HRP)-conjugated secondary antibody for 2 h at room temperature. After three additional washes with TBST, protein bands were visualized using an Enhanced Chemiluminescence (ECL) Kit (Beyotime, Shanghai, China). Band intensities were quantified using ImageJ software (National Institutes of Health, Bethesda, MD, USA).

### Statistical analysis

The experimental results were expressed as mean ± SEM. All experiments were repeated at least three times. Data analysis and figure creation were performed using GraphPad Prism 5.0 software. One-way analysis of variance was used to analyze differences among groups, followed by Dunnett post hoc comparisons when appropriate. *P* < 0.05 was judged to be statistically significant.

## RESULTS

### SIN alleviates histopathological damage in endometritis induced by *S. aureus*

**Fig 1:**
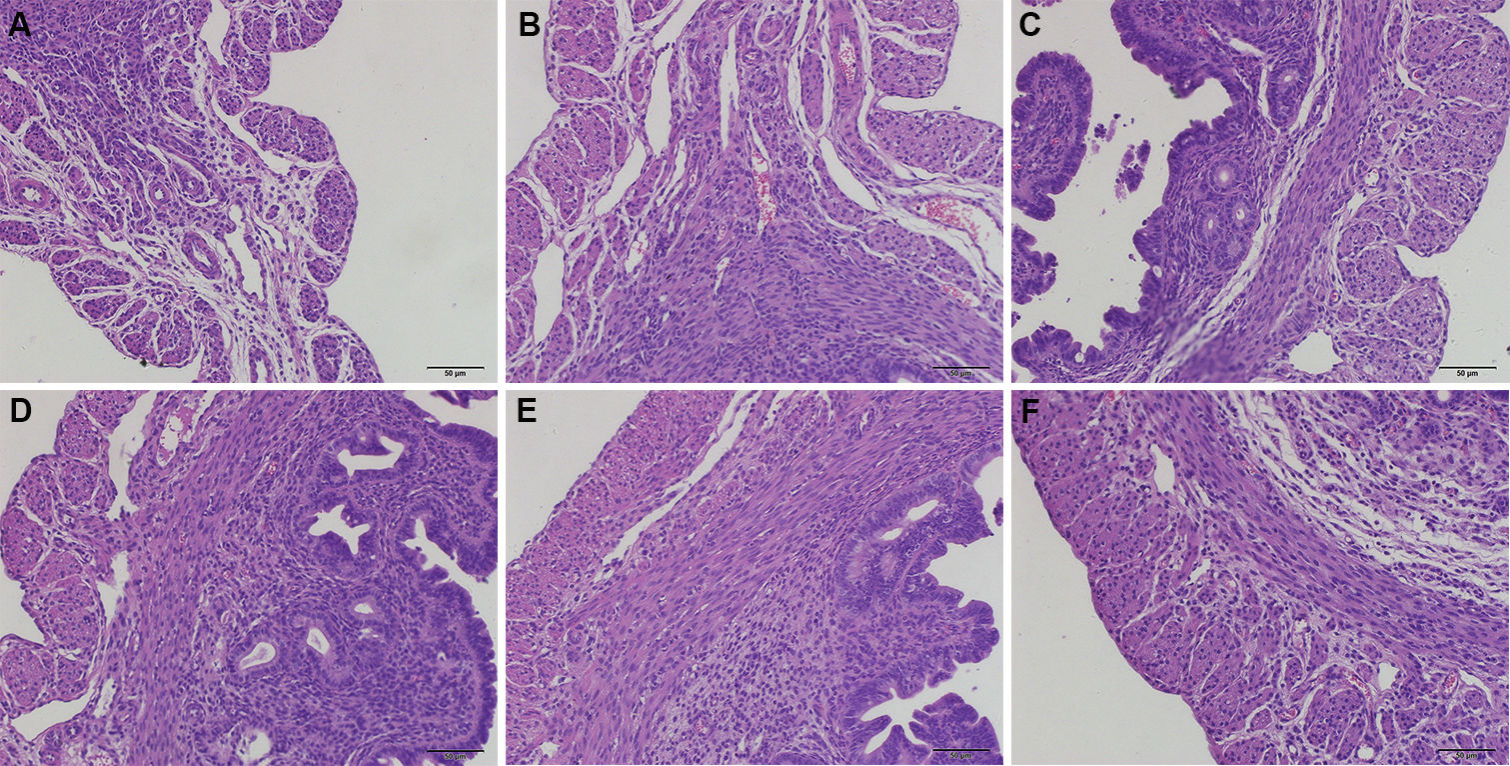
Effects of SIN on histopathological lesions in *S. aureus*-induced endometritis. Representative histopathological images of uterine tissues from different experimental groups: (**A**) Control group (no treatment); (**B**) SIN monotherapy group (administered SIN alone); (**C**) *S. aureus*-infected group (challenged with 1 × 10⁷ CFU/100 µL PBS); (**D–F**) SIN intervention groups (infected with *S. aureus* followed by treatment with 25 mg/kg, 50 mg/kg, or 100 mg/kg SIN, respectively).

The therapeutic efficacy of SIN against *S. aureus*-induced endometrial injury was evaluated using H&E staining. As shown in Fig. 1, no significant morphological alterations were observed in uterine tissues from mice treated with SIN alone compared to the control group. In contrast, intrauterine inoculation with 1 × 10^7^ CFU of *S. aureus* resulted in marked pathological changes, including tissue edema, disruption of normal tissue architecture, and extensive infiltration of inflammatory cells. Notably, different levels of SIN could alleviate uterine morphological damage caused by *S. aureus*.

### SIN alleviates MPO in endometritis tissue induced by *S. aureus*

**Fig 2:**
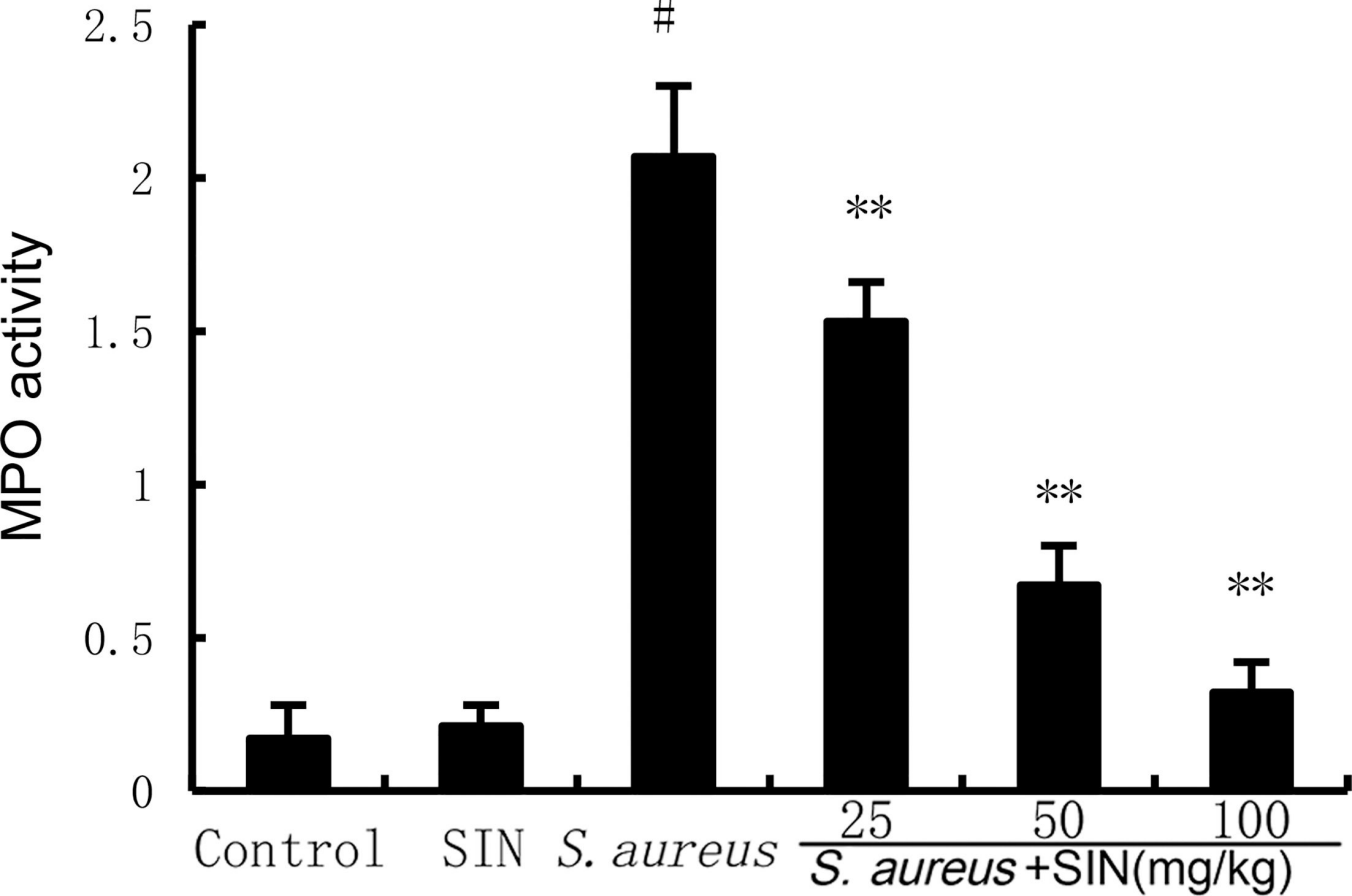
Impact of SIN on myeloperoxidase (MPO) activity in *S. aureus*-induced endometritis tissues. Data are expressed as mean ± standard error of the mean (SEM). Statistical significance: ^#^*P* < 0.05 vs the control group; **P < 0.05 vs the *S. aureus*-infected group.

MPO is a neutrophil-specific enzyme that constitutes approximately 5% of the cellular dry weight. Therefore, by detecting MPO activity, the neutrophils content and degree of inflammation in uterine tissue can be evaluated. As shown in Fig. 2, no significant difference in MPO activity was observed between the control group and the SIN-only treatment group (*P* > 0.05), indicating that SIN alone does not elicit an inflammatory response. In contrast, intrauterine inoculation with *S. aureus* resulted in a marked increase in MPO activity compared to the control group (*P* < 0.05), confirming the establishment of neutrophil-dominated inflammation. Notably, treatment with increasing doses of SIN significantly attenuated the *S. aureus*-induced elevation of MPO activity in a dose-dependent manner (*P* < 0.05).

### SIN alleviates the inflammatory response induced by *S. aureus*

**Fig 3:**
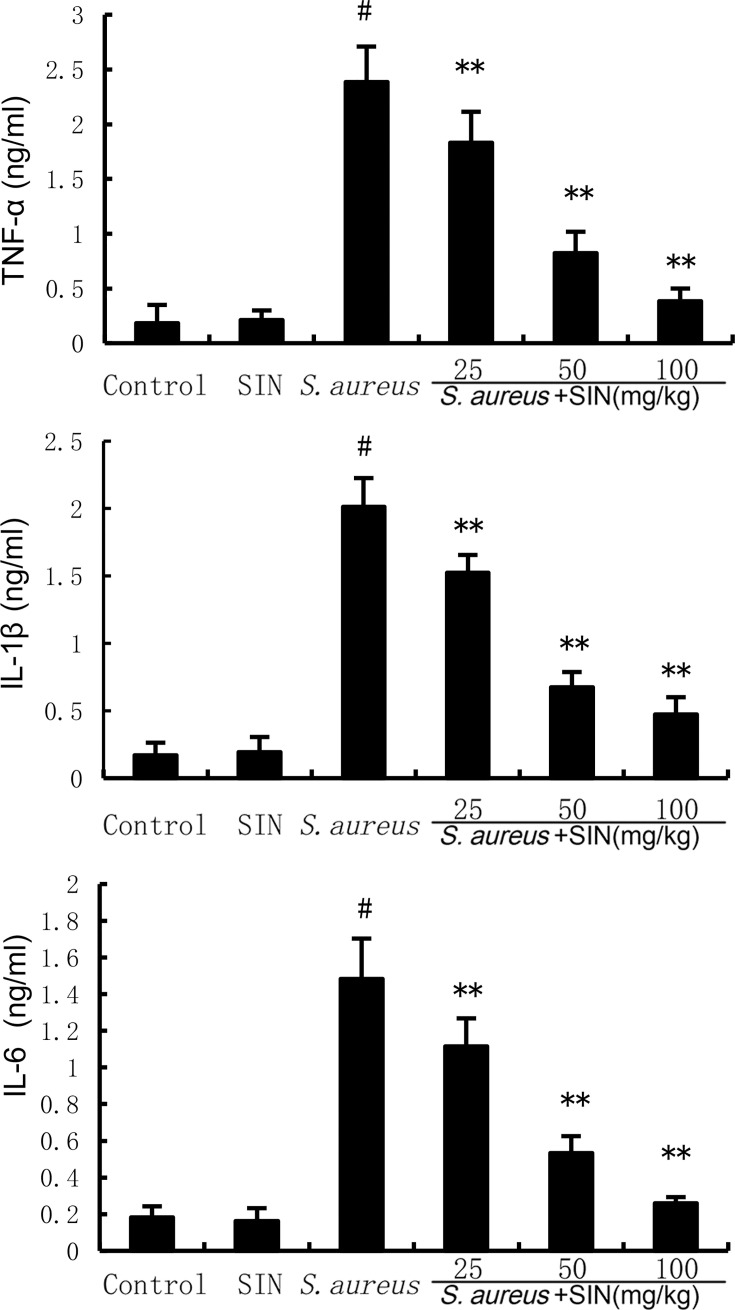
Suppressive effect of SIN on *S. aureus*-triggered inflammatory responses. Values are presented as mean ± SEM. ^#^*P* < 0.05 indicates a significant difference compared with the control group; **P < 0.05 indicates a significant difference compared with the *S. aureus*-infected group.

TNF-α, IL-1β, and IL-6 are key pro-inflammatory cytokines that orchestrate the inflammatory response. As shown in Fig. 3, no significant differences in the levels of TNF-α, IL-1β, or IL-6 were observed between the control group and the SIN-only treatment group (*P* > 0.05), indicating that SIN alone does not induce a pro-inflammatory response. In contrast, intrauterine inoculation with *S. aureus* significantly upregulated the production of these cytokines compared to the control group (*P* < 0.05). Notably, treatment with increasing doses of SIN markedly suppressed the *S. aureus*-induced elevation of TNF-α, IL-1β, and IL-6 levels in a dose-dependent manner (*P* < 0.05).

### SIN alleviates ferroptosis induced by *S. aureus*

**Fig 4:**
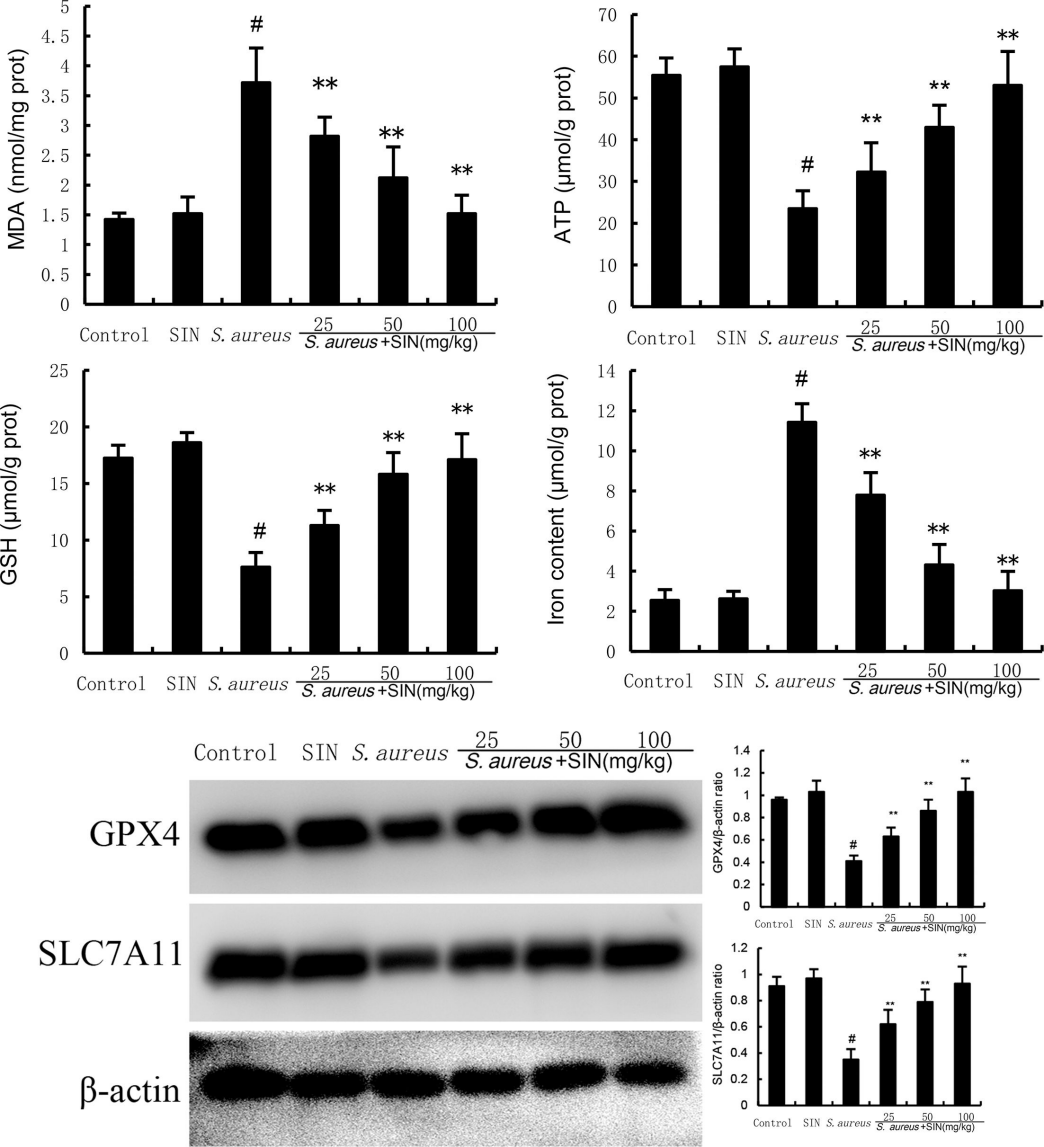
Protective role of SIN against *S. aureus*-induced ferroptosis in endometritis. Data are shown as mean ± SEM. ^#^*P* < 0.05 vs the control group; **P < 0.05 vs the *S. aureus*-infected group.

To evaluate the involvement of ferroptosis in *S. aureus*-induced endometritis and its modulation by SIN, we measured MDA, ATP, GSH, and iron content in uterine tissues. As shown in Fig. 4, treatment with SIN alone did not significantly alter these ferroptosis-related parameters compared to the control group (*P* > 0.05). In contrast, intrauterine inoculation with *S. aureus* significantly increased MDA and iron levels while decreasing ATP and GSH content (*P* < 0.05), indicating the induction of ferroptosis. Notably, administration of SIN dose-dependently reversed these *S. aureus*-induced alterations, as evidenced by reduced MDA and iron accumulation and restored ATP and GSH levels (*P* < 0.05). Subsequently, we detected the expression of key ferroptosis-regulatory proteins by western blotting. As shown in Fig. 4, *S. aureus* infection significantly downregulated the protein expression of GPX4 and SLC7A11 compared to the control group (*P* < 0.05). However, treatment with increasing concentrations of SIN markedly upregulated GPX4 and SLC7A11 expression in a dose-dependent manner relative to the *S. aureus*-alone group (*P* < 0.05). These results collectively suggest that SIN attenuates *S. aureus*-induced ferroptosis in endometrial tissue.

### SIN inhibited the activation of the NF-κB signaling pathway induced by *S. aureus*

**Fig 5:**
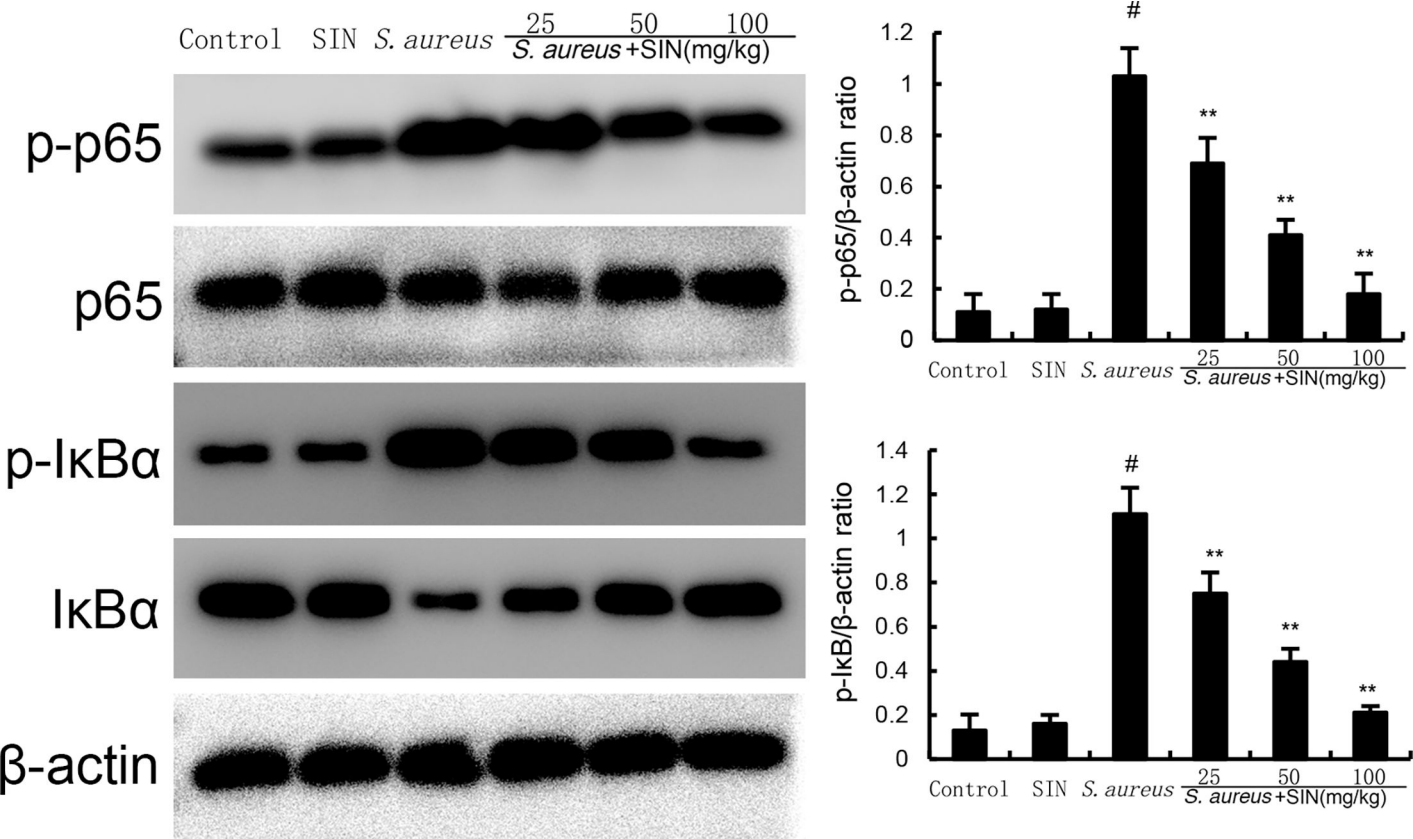
Inhibitory effect of SIN on *S. aureus*-induced activation of the NF-κB signaling pathway. Values are expressed as mean ± SEM. ^#^*P* < 0.05 denotes a significant difference compared with the control group; **P < 0.05 denotes a significant difference compared with the *S. aureus*-infected group.

To further elucidate the anti-inflammatory mechanism of SIN, we examined the activation status of the NF-κB signaling pathway in uterine tissues. As shown in Fig. 5, treatment with SIN alone did not significantly alter the p-p65 and p-IκBα levels compared to the control group (*P* > 0.05), indicating that SIN does not constitutively activate this pathway. In contrast, intrauterine inoculation with *S. aureus* significantly upregulated the expression of p-p65 and p-IκBα relative to the control group (*P* < 0.05), confirming the induction of NF-κB-mediated inflammatory signaling. Notably, administration of increasing doses of SIN markedly suppressed the *S. aureus*-induced phosphorylation of p65 and IκBα in a dose-dependent manner (*P* < 0.05). These results suggest that SIN attenuates the inflammatory response in endometritis by inhibiting the activation of the NF-κB signaling pathway.

### SIN inhibited the expression of NLRP3 induced by *S. aureus*

**Fig 6:**
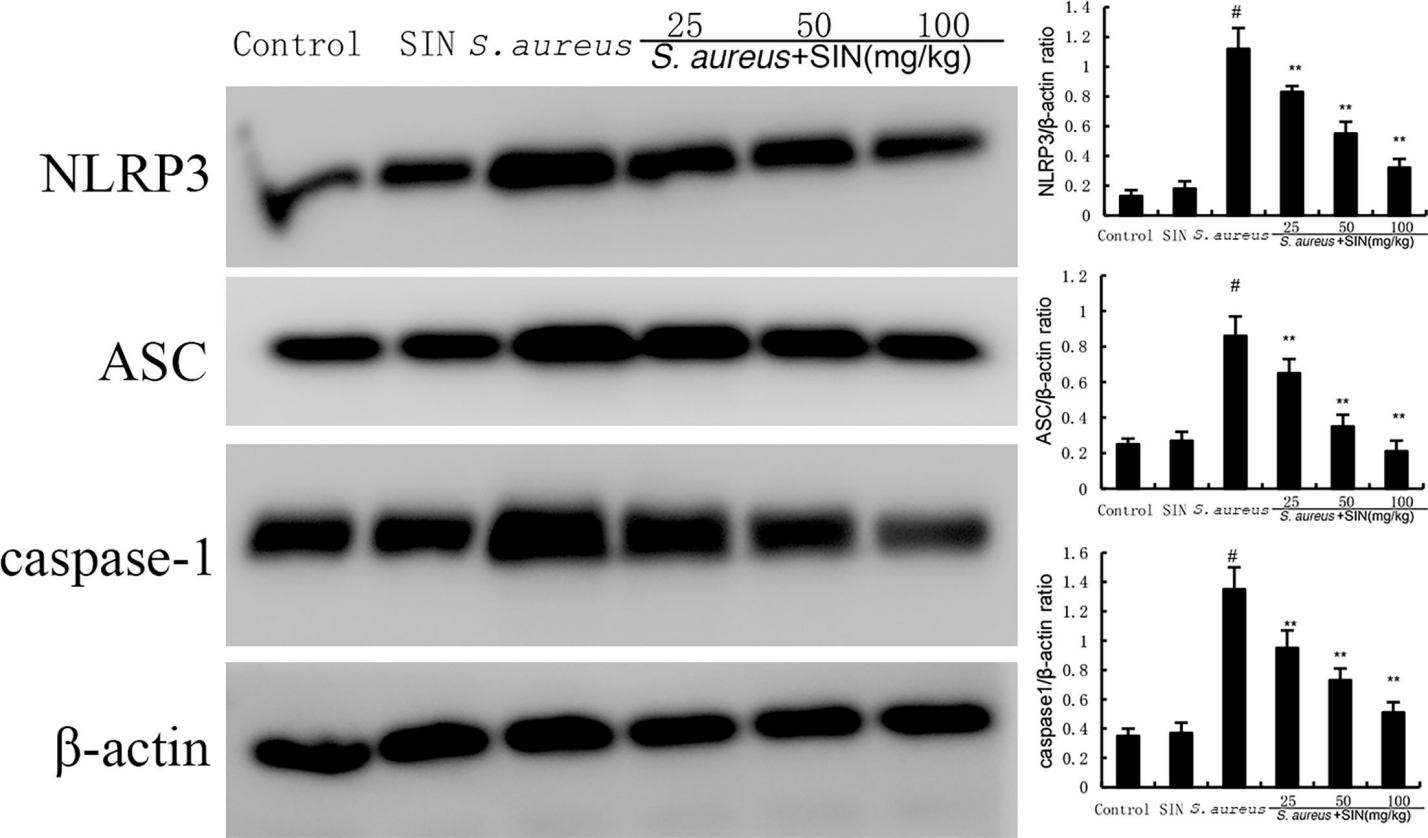
Downregulation of *S. aureus*-induced NLRP3 expression by SIN. Data are presented as mean ± SEM. ^#^*P* < 0.05 vs the control group; **P < 0.05 vs the *S. aureus*-infected group.

To investigate whether SIN modulates the NLRP3 inflammasome, we examined the protein expression of NLRP3 and apoptosis-associated speck-like protein (ASC) in uterine tissues. As shown in Fig. 6, intrauterine inoculation with *S. aureus* significantly upregulated the expression of both NLRP3 and ASC compared to the control group (*P* < 0.05), indicating activation of the NLRP3 inflammasome complex. In contrast, treatment with increasing doses of SIN markedly suppressed the *S. aureus*-induced elevation of NLRP3 and ASC protein levels in a dose-dependent manner (*P* < 0.05). These findings suggest that the anti-inflammatory effect of SIN in *S. aureus*-induced endometritis may involve inhibition of NLRP3 inflammasome activation.

### SIN inhibited the expression of P2X7 induced by *S. aureus*

**Fig 7:**
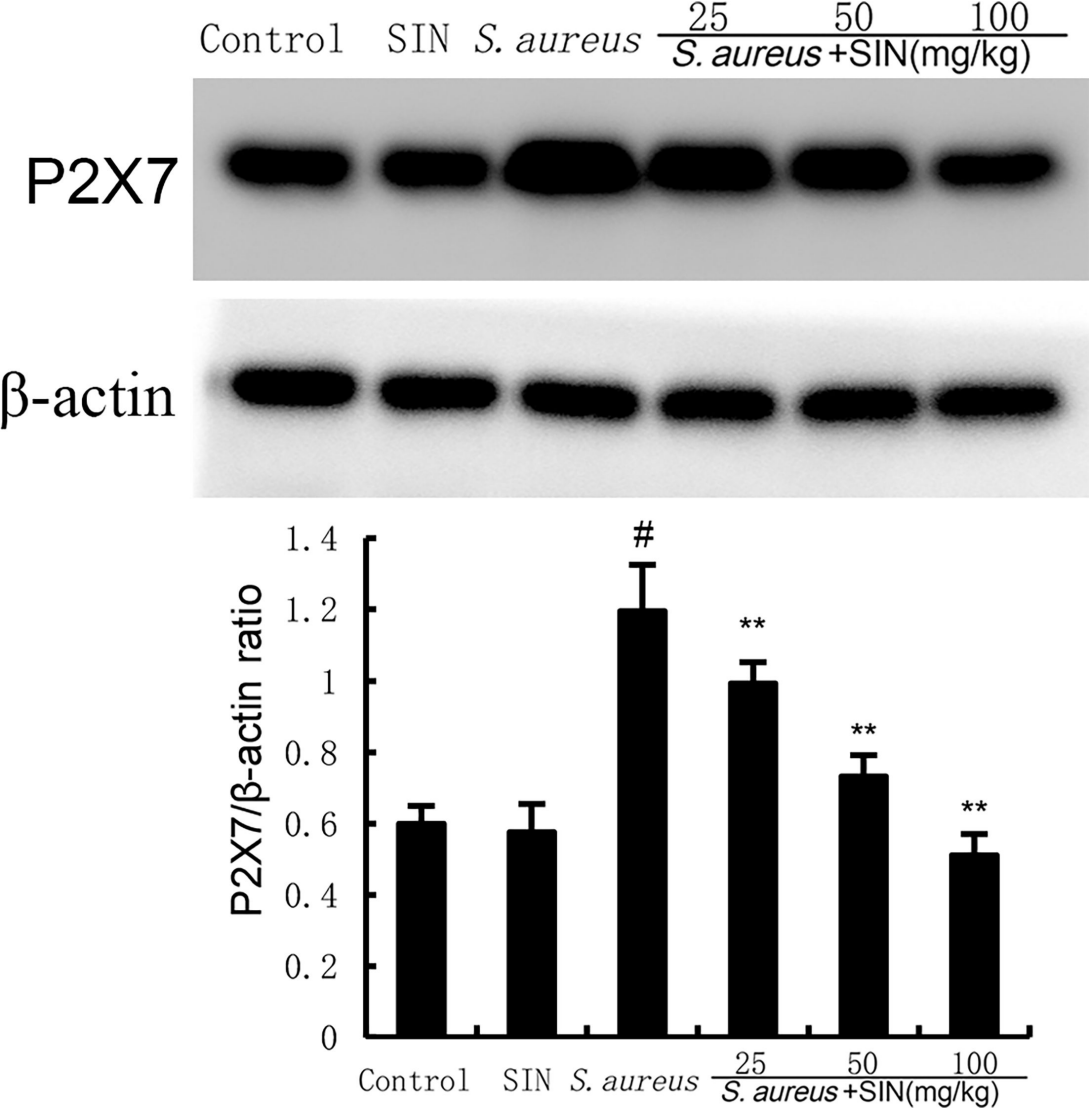
SIN reduces *S. aureus*-induced P2X7 expression in endometritis tissues. Values are shown as mean ± SEM. ^#^*P* < 0.05 is considered significantly different from the control group; **P < 0.05 is considered significantly different from the *S. aureus*-infected group.

We next examined P2X7 expression in uterine tissues. As shown in Fig. 7, intrauterine inoculation with *S. aureus* significantly upregulated P2X7 protein expression compared to the control group (*P* < 0.05). In contrast, treatment with increasing doses of SIN markedly suppressed the *S. aureus*-induced elevation of P2X7 expression in a dose-dependent manner (*P* < 0.05). These results suggest that SIN may attenuate NLRP3 inflammasome activation by downregulating P2X7 receptor expression.

## DISCUSSION

Endometritis imposes substantial economic burdens on the livestock industry, and widespread use of antibiotics has raised concerns regarding antimicrobial resistance and potential adverse effects. Therefore, there is an urgent need to identify effective alternatives with favorable safety profiles. SIN, a bioactive alkaloid purified from *S. acutum*, has attracted considerable attention due to its well-documented anti-inflammatory and immunomodulatory properties, coupled with a favorable safety profile (24). Emerging evidence suggests that the P2X7 receptor contributes to inflammatory responses through the regulation of ferroptosis (25). The present study demonstrated that SIN attenuates *S. aureus*-induced endometritis by inhibiting the P2X7 receptor and suppressing ferroptosis. These results support the potential of SIN as a promising non-antibiotic therapeutic strategy for the treatment of endometritis.

Endometritis in livestock disrupts the estrus cycle, reduces milk yield, and delays conception, ultimately resulting in substantial economic losses (26). The etiology of endometritis is multifactorial, with pathogenic microbial invasion recognized as the primary causative factor. Bacteria such as *Escherichia coli*, *S. aureus*, and *Pseudomonas aeruginosa* can induce endometritis through the secretion of endotoxins, including lipopolysaccharide (LPS) (27). Although antibiotic therapy has historically played a pivotal role in the initial management of endometritis, its widespread application is increasingly constrained by the emergence of antimicrobial resistance and concerns regarding drug residues (3). Extracts derived from traditional Chinese medicine have attracted increasing attention as potential alternatives for the prevention and treatment of endometritis, owing to their potent bioactivities and low potential for inducing drug resistance. SIN, an alkaloid monomer extracted from the roots and stems of *S. acutum*, has been the subject of extensive pharmacological investigation. Accumulating experimental evidence indicates that SIN exerts anti-inflammatory effects by downregulating pro-inflammatory cytokines and upregulating anti-inflammatory cytokines in the serum of model animals (28). In addition, SIN has been shown to modulate T-lymphocyte activity, contributing to its therapeutic efficacy in immune-mediated disorders through anti-inflammatory and analgesic mechanisms (29). Clinical studies further support these findings, demonstrating that SIN reduces the levels of pro-inflammatory cytokines such as TNF-α in the synovial fluid of patients with rheumatoid arthritis and significantly alleviates the symptoms of knee arthritis (30).

Similarly, the present study found that SIN treatment significantly reversed the *S. aureus*-induced elevation of pro-inflammatory cytokines, including TNF-α, IL-1β, and IL-6, in a mouse model of endometritis, indicating that SIN effectively suppresses the inflammatory response associated with this condition. Previous research has reported that SIN alleviates rheumatoid arthritis by modulating mononuclear cell proliferation, inflammatory cytokine production, and regulatory T-cell function in peripheral blood (31). In line with this immunomodulatory role, our results further revealed that SIN reduced MPO activity in uterine tissues, indicating that SIN may attenuate endometritis, at least in part, through regulation of the immune response.

Ferroptosis is a recently characterized form of regulated cell death driven by iron-dependent lipid peroxidation. Distinct from other cell death modalities, ferroptosis is classified as a subtype of regulatory necrosis and exhibits greater immunogenicity than apoptosis (32). Emerging evidence has implicated ferroptosis in host tissue damage resulting from bacterial infection. For instance, infection with Gram-negative bacteria such as *P. aeruginosa* has been shown to induce chronic inflammation in human bronchial epithelial cells, accompanied by lipid peroxidation and subsequent ferroptotic cell death (33). In line with these findings, the present study demonstrated that *S. aureus* infection significantly increased MDA and iron levels while reducing ATP and GSH content in uterine tissues, indicating that *S. aureus* infection triggers ferroptosis in the context of endometritis. Previous studies have found that ferroptosis can lead to a decrease in ATP content (34), whereas inhibiting ferroptosis can eliminate the trend of decreased ATP content (35), indicating that ATP plays an important indicative role in the process of ferroptosis. In mice, pharmacological inhibition of ferroptosis has been shown to alleviate dextran sulfate sodium (DSS)-induced colitis, an effect attributed to the attenuation of endoplasmic reticulum stress and the upregulation of GPX4 expression (36). Ferroptosis has also been implicated as a potential trigger in the pathogenesis of fatty liver disease. In mice with nonalcoholic steatohepatitis (NASH), administration of ferroptosis inducers was found to promote the release of pro-inflammatory cytokines, exacerbating hepatic inflammation (37). Conversely, treatment with ferroptosis inhibitors suppressed lipid peroxidation in the liver and significantly reduced both inflammatory cytokine levels and immune cell infiltration (38). Recent studies have found that inhibition of ferroptosis exerts a protective effect against LPS-induced endometritis (39). Further research results demonstrated that nuciferine alleviates LPS-induced endometritis by regulating ferroptosis via the MYD88 signaling pathway (40). Consistent with these findings, the present study revealed that SIN attenuated ferroptosis induced by *S. aureus* infection. Mechanistically, SIN treatment significantly suppressed the expression of GPX4 and SLC7A11, key regulators of ferroptosis, suggesting that SIN alleviates endometritis through inhibition of ferroptosis.

The P2X7 receptor is a ligand-gated ion channel belonging to the P2 purinergic receptor family. It is widely expressed in various tissues and plays a critical role in modulating cellular physiological functions, thereby influencing the pathogenesis and progression of multiple diseases (41). Accumulating evidence has demonstrated that P2X7 receptor activation potently triggers inflammatory responses. Mechanistically, P2X7 activation promotes the release of pro-inflammatory cytokines by stimulating the NLRP3 inflammasome and caspase-1/3 signaling pathways (42). Upon activation, P2X7 induces the efflux of K^+^ and the influx of Ca²^+^ and Na^+^, leading to NF-κB-mediated caspase-1 activation, which subsequently cleaves pro-IL-1β into its mature, biologically active form (43). Furthermore, sustained activation of P2X7 results in the formation of non-selective membrane macropores, facilitating the extracellular release of mature IL-1β and thereby amplifying the local inflammatory response (44). P2X7 knockdown has been demonstrated to suppress ferroptosis by attenuating lipid peroxidation *via* inhibition of the MAPK/ERK signaling pathway (45). Additionally, P2X7 deficiency significantly ameliorated cardiac ferroptosis in angiotensin II-induced mice (46). Our findings suggest that SIN inhibits the expression of P2X7 and NF-κB pathway-related proteins, implying that SIN may alleviate uterine inflammation by suppressing ferroptosis through the P2X7 signaling pathway.

In conclusion, the present study demonstrates that SIN alleviates *S. aureus*-induced endometritis in mice. The underlying mechanism appears to involve attenuation of ferroptosis through modulation of the P2X7 receptor/NLRP3 signaling pathway. These findings provide a theoretical basis for the potential application of SIN as an adjunctive therapeutic agent in the clinical management of endometritis.

## Data Availability

The data used to support the findings of this study are available from the corresponding author upon request.
